# Supplementary material for: Long-Term Suppression of c-Jun and nNOS Preserves Ultrastructural Features of Lower Motor Neurons and Forelimb Function after Brachial Plexus Roots Avulsion
Source: Cells. 2021 Jun 28;10(7):1614. doi: 10.3390/cells10071614 (PMC8307634; doi:10.3390/cells10071614)
Supplement: Supplementary file 1 [file cells-10-01614-s001.zip › cells-1194941-supplementary.pdf]

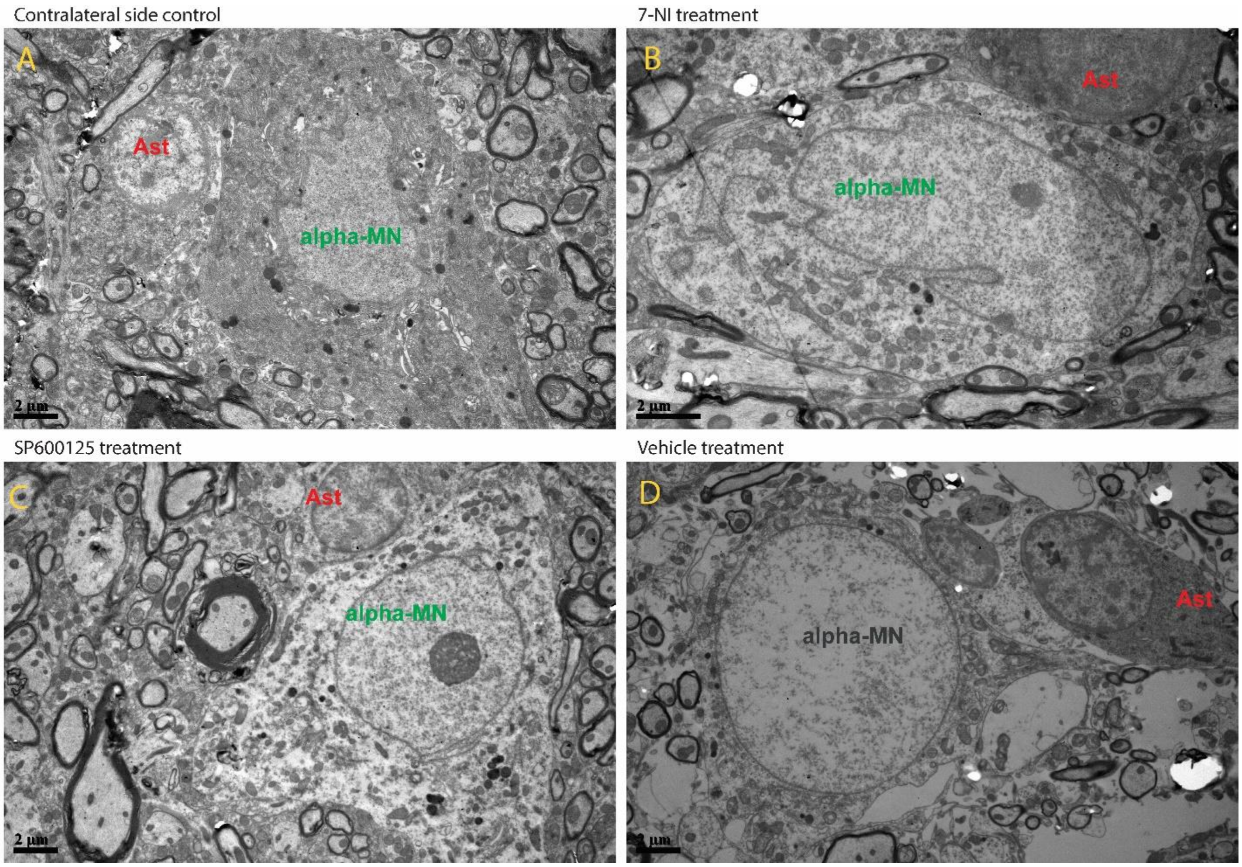


**Figure S1.** The range of motor neuro-astrocyte interaction in different groups. There was normal α-motor neuron-astrocyte interaction in the 7-nitroindazole and Sp600125 treated rats. Degenerative α-motor neurons of vehicle treated rats were frequently fund to be associated with astrocytes exhibiting phagocytosis (alpha motor neuron-alpha-MN, Ast-astrocyte). Scale bar = 2µm. X3800.

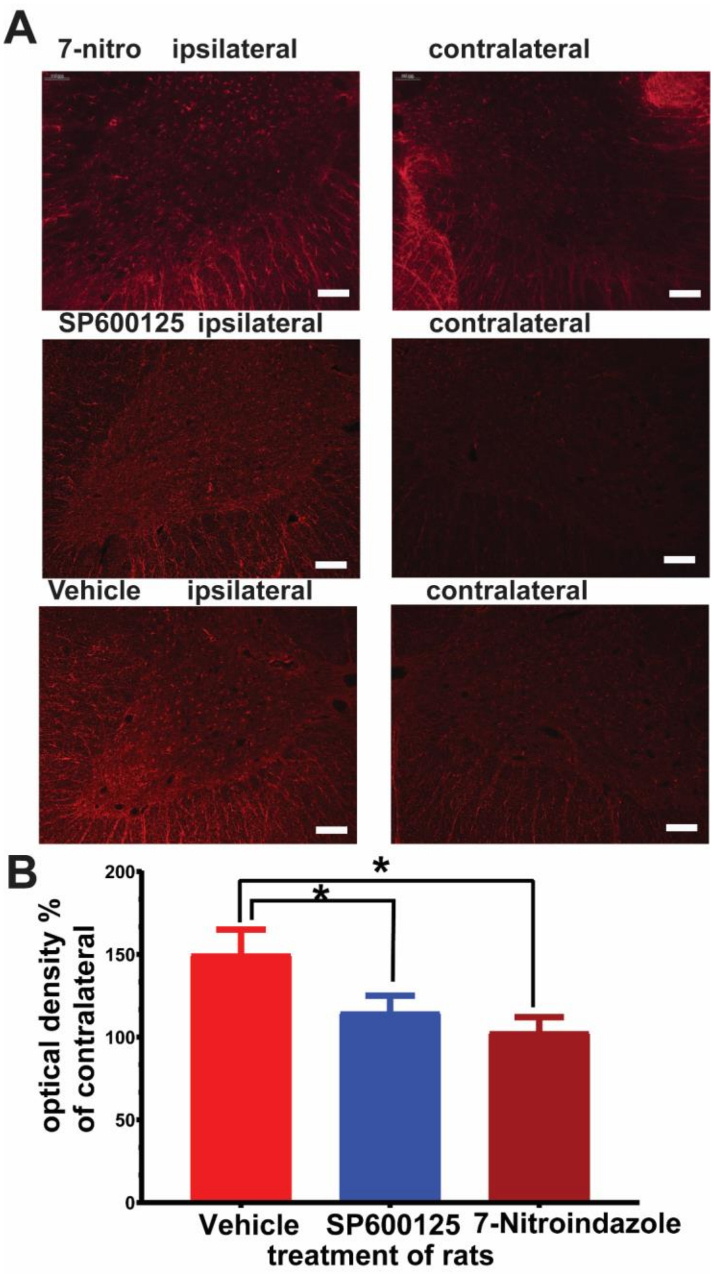


Figure S2 A: 4 Immunofluorescence staining to detect the expression of glial fibrillary acidic protein (GFAP) positive astrocytes (red) at 6 months after avulsion. B: optical density expressed as a percentage of the contralateral side. Vehicle treated rats exhibited residual astrocytic activation at 6 months after avulsion. Scale bar = 100μm. Mean±SD, * denotes $P<0.05$ Anova with Tukey post hoc test.

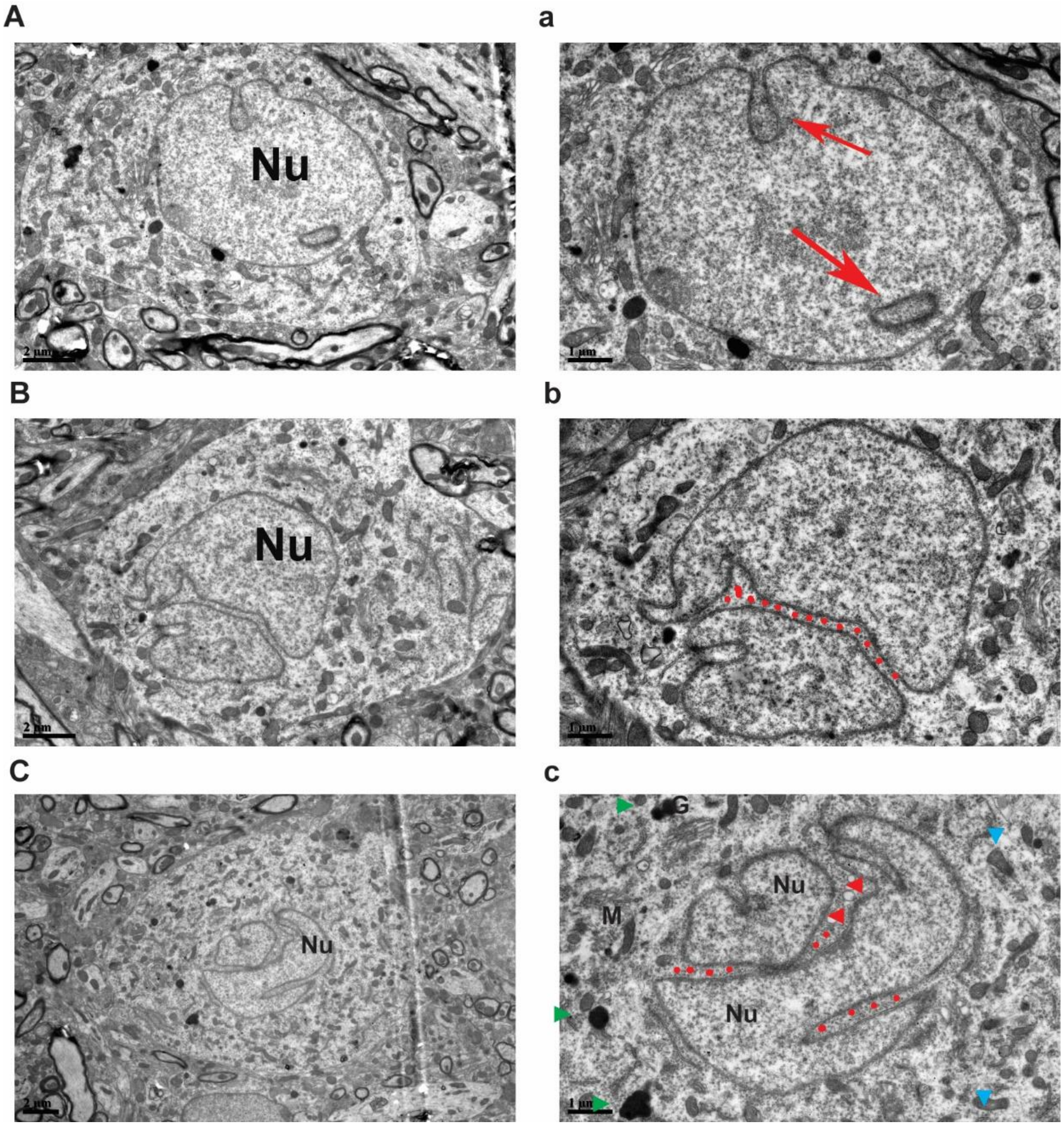


**Figure S3.** Peculiar nuclei morphologies of the 7-nitroindazole treated rats. There was a continuum of reticularization of the nucleus (spaces indicated by red dots) in the 7-nitroindazole treated rats. A is showing the mildest form (enlarged in a, red arrow show membrane-bound vesicle inside the nucleus) while C (enlarged c) depicts the highly reticularized nucleus of the representative α-motor neuron. Scale bar = 1μm and 2μm. Nu- nucleus; blue arrowhead-mitochondrion (M); G-Golgi apparatus; red arrowheads-cleft between nucleus masses containing vacuoles. X3800, X5800.
